# Algorithms for Empathy: Using Machine Learning to Categorize Common Empathetic Traits Across Professional and Peer-Based Conversations

**DOI:** 10.7759/cureus.57719

**Published:** 2024-04-06

**Authors:** Scott Provence, Alyssa A Forcehimes

**Affiliations:** 1 Founder, Versant Metrics, Duvall, USA; 2 Board Member, Versant Metrics, Paradise Valley, USA

**Keywords:** ai-based education, language and communication, education, empathy, artificial intelligence, machine learning

## Abstract

Introduction

In this article, we describe our creation of a machine-learning model that uses a combination of rule-based and natural language processing (NLP) algorithms. We show how this “Empathy Algorithm” was developed and how its results compare to three datasets of professional counseling and peer-led conversations.

Methods

These conversation datasets were rated by people with varying degrees of empathetic expertise (from counselors to student volunteers) and labeled as either low- or high-quality empathy. Our methodology involved running both these “low-empathy” and “high-empathy” conversations through our algorithm and then looking for a correlation between conversations labeled “high empathy” and an increased presence of six empathy skills flagged by our algorithm.

Results

We found positive correlations between four of the six skills that our algorithm measures (i.e., four empathizing skills showed up the same or more in each of the “high-empathy” conversations within the three datasets). This suggests that certain empathizing skills are not only consistently present in effective conversations but also quantifiable enough to be measured by today’s machine-learning models.

Conclusion

While limitations of language, binary classifications, and non-verbal cues remain as opportunities for further development, using algorithms to objectively assess empathic skills represents an important step to improve client outcomes and refine communication practices for today’s healthcare professionals.

## Introduction

Effective and empathetic communication is a linchpin for positive client outcomes across various professional domains. While empathy is often defined as the ability to understand what others feel, empathic communication is more about one's ability to respond to an expression in a way that conveys understanding in a respectful and empowering way. The nuanced nature of empathy, coupled with the inherent challenges in its definition and teachability, has prompted a quest for innovative approaches to its assessment [1,2]. This, combined with recent strides in machine learning (ML), offers a unique opportunity to bridge the gap in helping define and objectively evaluate what it means to respond with empathy.

While much of the concepts of artificial intelligence (AI) and ML (a branch of AI) fall outside the scope of this article, there is a particular subset of ML essential to the scope of this study. As a whole, ML focuses on building systems that analyze large amounts of data to make predictions, classifications, or decisions. The subset of ML that is foundational in our work is known as natural language processing (NLP). NLP is often used for tasks, such as translation, speech recognition, and sentiment analysis. In our work, we rely on the capabilities of NLP specifically as it relates to "supervised" ML, in which text from labeled datasets is used to build patterns that predictively classify inputs from new conversations.

The conversations held between a professional and a client and the interpersonal skill of empathy, in particular, have been extensively researched within the fields of healthcare and psychotherapy, where results have shown a causal relationship between provider empathy and positive client outcomes [3-11]. However, the elusive nature of empathy, both in definition and teachability, poses a challenge [12]. The difficulty in providing objective feedback in a timely manner - the best way to increase skills in empathic behavior - further complicates its assessment [2,13,14].

Advancements in the field of ML are creating new opportunities for objectively evaluating complex human qualities, including empathy [14-16]. In fact, some argue that machine learning algorithms can now analyze psychotherapy sessions with a level of consistency comparable to human counterparts [13]. This breakthrough presents an opportunity to bridge the gap in assessing empathy by creating tools that offer objective, real-time feedback to counselors, physicians, and professionals in various communication-intensive domains.

While certain indicators of empathy lend themselves to quantification, such as tracking the time spent listening versus speaking, others, like reflective statements, demand a more nuanced analysis [1]. In response to this challenge, we built a program using a combination of rule-based and NLP algorithms that would evaluate the level of empathy in a participant's response to a speaker.

This article introduces the Empathy Algorithm: a machine-learning model crafted by a collaboration of clinical psychologists and behavior change experts. Drawing inspiration from evidence-based counseling methods, particularly motivational interviewing (MI), a client-centered counseling style [17], this algorithm evaluates the text of any two-person conversation and assesses the level of empathic response using six distinct categories. Rule-based analysis of each category, which includes reflections, suspension of the listener's perspective, affirmations, open questions, avoidance of directing, and time spent listening versus speaking, collectively form the algorithm's foundation.

In this article, we delve into our work in developing and testing the Empathy Algorithm, outlining its underlying mechanisms and discussing its potential impact on diverse fields. The focal point of our study is to assess how well our algorithm performs in comparison to human evaluations across a spectrum of conversations.

## Materials and methods

In order to test the efficacy of the Empathy Algorithm, we evaluated 187 conversations (10,681 total utterances) sourced from three diverse datasets [18-20]. Each of these datasets was evaluated by human annotators with different backgrounds and familiarity levels with empathic and professional counseling practices. Our goal was to determine if the Empathy Algorithm scoring would consistently agree with the designations of “low” and “high” empathy assigned to these conversations by their human evaluators.

This study was designed as a two-phase evaluation of an empathy algorithm that analyzes associations between human-scored conversations and the presence of empathy skills as classified by pre-programmed rules and machine-learning models. The Empathy Algorithm uses a combination of decision-tree logic and NLP techniques to arrive at its classifications. The decision-tree logic consists of both branching and binary criteria. In addition to this rule-based logic, the algorithm also uses vector-matching NLP models to analyze linguistic patterns to find semantic similarities between text segments. This combination of decision-tree logic and advanced NLP models allowed us to best track each answer the algorithm arrives at.

Many ethical considerations need to be accounted for in studies such as this. Not only were the human-conversation datasets used in this study made publicly available, but they were also already de-identified. Thus, while our own algorithm also did not store conversation data, the fact that original datasets had already been de-identified provided all subjects with a secondary layer of protection. Finally, because we used branching logic alongside our ML models, we have more transparency into our algorithm’s decision-making processes, an important feature when considering future studies and real-world impact.

Our primary method for statistical analysis was to simply quantify the occurrence of each empathizing skill across all of our three conversation datasets. For each dataset, the algorithm evaluated each professional- or peer-based response as it occurred in the conversation. The fact that the same decision-tree rules and ML model were used across all datasets helped ensure consistency and accuracy in our data collection. For our comparative analysis, we calculated the average frequency of occurrence for each of our six empathizing skills, which we then normalized to represent as a percentage of each conversation. Finally, we curated a visualization of this analysis using bar charts, created in spreadsheet software, namely, Microsoft Excel (version 16.78.3, Microsoft Corporation, United States) and Google Sheets (Google LLC, United States).

Study phase 1: category construction

In constructing the Empathy Algorithm, our team of clinical psychologists, coders, and behavior change experts drew inspiration from evidence-based counseling methods, particularly MI [17]. To this method, we add other well-studied communication skills, including perspective-taking, client affirmations, and using restraint around instruction-giving and other forms of advice [15,21,22]. Our algorithm was designed to collectively analyze these categories, each reflective of crucial aspects of empathic communication. Our categories of evaluation include the following:

Offering Reflections

Effective reflections involve making insightful guesses about the speaker's intended meaning, fostering a deeper understanding of their perspective.

Suspending One's Own Perspective

The goal of this skill is to stay focused on the speaker's viewpoint. To excel in this category, participants must demonstrate that they are setting aside their point of view, redirecting the conversation to center on the speaker's perspective.

Getting Affirmations for Being On Track

Following an empathetic expression, participants should actively listen for any affirmations from the speaker, which can confirm that the participant’s response aligned with the speaker’s intended message.

Asking Open Questions

Open-ended questions are those that cannot be answered with a simple "Yes" or "No" response. They encourage the speaker to elaborate, clarify, or provide deeper insights.

Avoiding Minimizing or Directing

This category emphasizes the challenge of refraining from offering unsolicited opinions or advice, allowing the speaker to express themselves without interference. It also addresses the tendency to minimize emotions or jump too quickly into “problem-solving.”

Listening More than Speaking

Actively prioritizing listening over speaking fosters an environment where the other person feels the freedom to speak their mind. It enables a deeper connection by giving space for the speaker's narrative to unfold.

**Figure 1:**
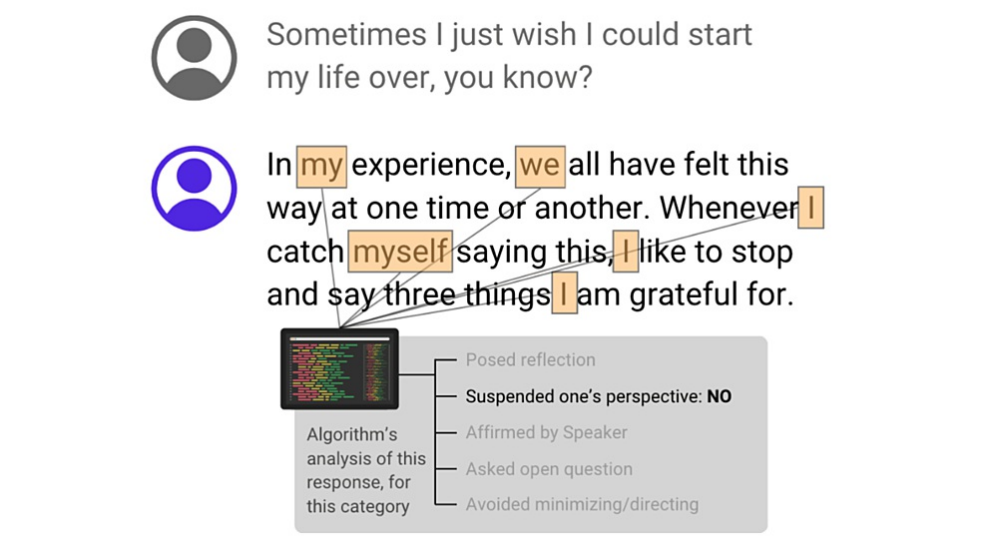
A simplified example of how the Empathy Algorithm analyzes responses

These empathizing categories collectively form the foundation of the Empathy Algorithm, guiding its evaluation of conversations by assessing the presence and efficacy of these critical empathic skills. Figure 1 demonstrates a simplified example of how the Empathy Algorithm analyzes a response to determine a categorical rating.

While the details of how Empathy Algorithm assesses each of these categories are proprietary, we acknowledge the importance of transparency and knowledge-sharing in the scientific community. To facilitate replication and the advancement of this work, our goal in the above explanations is to offer enough information about our categories and methodology so that others can further explore and add to this study.

Study phase 2: comparing the algorithm against human evaluators

Next, our research aimed to rigorously test the effectiveness of the Empathy Algorithm against human evaluations of empathic communication. However, since there is no one population or certification for the ability to assess empathy, our aim was to compare our outputs against as broad a spectrum of human evaluators as possible.

To test the algorithm, we had it assess a total of 187 conversations sourced from three distinct datasets, each of which had been previously rated by an independent group of evaluators. Because the sampling technique and inclusion/exclusion criteria were not fully detailed in some of the original datasets provided, we have included what criteria and techniques were mentioned, and have drawn attention to this potential selection/sampling variable within our review of limitations.

Dataset 1: Text-Based Dialogues, Evaluated by Non-professional, High-EQ Volunteers

Our first dataset [18] was a subset of text-based conversations that had been further refined by a panel of 10 highly empathetic volunteers, as determined by an EQ (Emotional Quotient) test in which volunteers had to surpass a benchmark score. These volunteers were sourced from online communities and students of Instituto Tecnológico y de Estudios Superiores de Monterrey. The volunteers evaluated conversations using a five-point Likert scale, assigning an "Empathy Score" ranging from 1 (not empathetic at all) to 5 (very much empathetic). In order to best mirror the binary “low” and “high” evaluations of our other two datasets, we only used conversations from this dataset that had scored either a “1” or a “5,” representing the interactions that were graded the very least and very most empathetic, respectively.

Dataset 2: Video-Based Dialogues Evaluated by Counseling Professionals

This dataset [19] consisted of transcribed demonstrations of high- and low-quality MI from video-sharing platforms (YouTube and Vimeo). These conversations were assigned a label of "high-quality" or "low-quality" MI and were further evaluated and annotated line by line by 10 therapists who are members of the Motivational Interviewing Network of Trainers (MINT), an international organization and widely recognized authority in best practices in MI [21]. Specific conversations of varied length and quality were also evaluated by all annotators to determine inter-annotator agreement (IAA). The authors who initially refined this dataset study used Fleiss’ kappa to measure the IAA between the 10 annotators, recording 0.74 (substantial agreement) for their labels of the behavioral code assigned.

Dataset 3: Video-Based Dialogues Conducted and Collected by Motivational Interviewing Treatment Integrity (MITI) Experts

This dataset contained coded transcripts of high- and low-quality MI, further evaluated by students trained to fidelity in the Motivational Interviewing Treatment Integrity (MITI) coding system [20]. This dataset was made available from the Center of Alcohol, Substance Use, and Addiction (CASAA) center at the University of New Mexico. Conversations were coded by individuals who underwent a rigorous training program to ensure adherence to MI principles. As with the previous dataset, rather than matching our algorithm to the line-by-line MITI codes, we kept our evaluation focused on each conversation’s overall label of “high-” or “low-quality" MI. For this dataset, these high/low labels were determined by the trainers and developers of the MITI coding instrument. These MITI experts were often the ones participating in the high-quality MI dialogues themselves. Therefore, we viewed this dataset as representing some of the highest fidelity to the practice of MI.

The conversations within each of these three datasets have thus been rated by varying degrees of empathetic expertise (e.g., professional counselors, developers of the MITI coding instrument, etc.) and separated into low- and high-quality ratings. For Dataset 1, this was low and high "empathy,” and for Datasets 2 and 3, this was low- and high-quality MI. While MI represents a complex and diverse set of counseling skills (of which empathy plays one key role), for the purpose of this study, we treated the low/high ratings for all three datasets as generally equivalent to each other.

**Figure 2:**
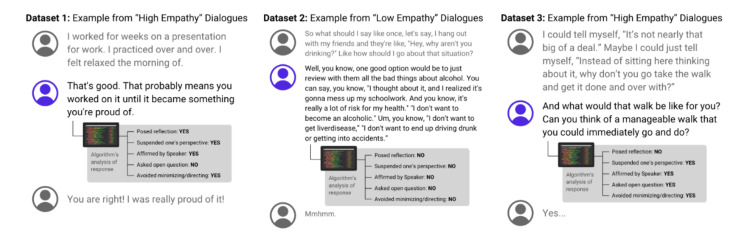
Example ratings of single responses within “high-empathy” and “low-empathy” labeled dialogues

To achieve our goal of comparative analysis for our Empathy Algorithm, our research involved running these “low-empathy” and “high-empathy” conversations through our Empathy Algorithm. We then compared how our algorithm scored each conversation against the "low-" and "high-quality" labels initially assigned by human evaluators, as demonstrated in Figure 2. Our aim was to determine which categories of our Empathy Algorithm were most aligned with these diverse, human-based interpretations of empathy.

## Results

To assess the effectiveness of the Empathy Algorithm, we conducted a comprehensive analysis comparing human-based ratings of low and high empathy with each of the category-specific ratings returned by our algorithm. This approach allowed us to discern patterns and identify specific empathy skills that stood out in each dataset, shedding light on the nuances of human evaluators' perceptions of empathy. The summary data can be found in Table 1.

**Table 1:** Category-specific ratings of the Empathy Algorithm across three conversational datasets EQ: Emotional Quotient, MI

DATASET 1: High-EQ volunteers	"Low" empathy	"High" empathy	% delta
% of time listening	not counted, due to the text-only nature of the dataset		
Posed reflections	0%	20%	20
Affirmed by the speaker	8%	22%	14
Suspended perspective	8%	16%	8
Open questions	8%	7%	1
No minimizing/directing	69%	70%	-1
DATASET 2: MINT-member therapists	"Low" MI	"High" MI	% Delta
% of time listening	36%	48%	12
Posed reflections	7%	10%	3
Affirmed by the speaker	42%	27%	-15
Suspended perspective	15%	20%	5
Open questions	14%	9%	-5
No minimizing/directing	41%	44%	3
DATASET 3: MI founders and experts	"Low" MI	"High" MI	% Delta
% of time listening	50%	57%	7
Posed reflections	14%	22%	8
Affirmed by the speaker	53%	53%	0
Suspended perspective	29%	28%	-1
Open questions	10%	9%	-1
No minimizing/directing	44%	47%	3

Based on our analysis and each set of evaluators' perceptions of empathy, we drew the following conclusions.

High-EQ volunteers (Dataset 1)

For conversations evaluated by non-trained but empathetic volunteers, the Empathy Algorithm’s categorizations of their “high-” and “low-”empathy conversations revealed some intriguing insights. This group of evaluators clearly saw a person as being more empathetic when they made more reflective statements. This one change showed the highest increase in all categories across all datasets: a 20% increase in the use of reflection statements for highly empathetic conversations.

**Figure 3:**
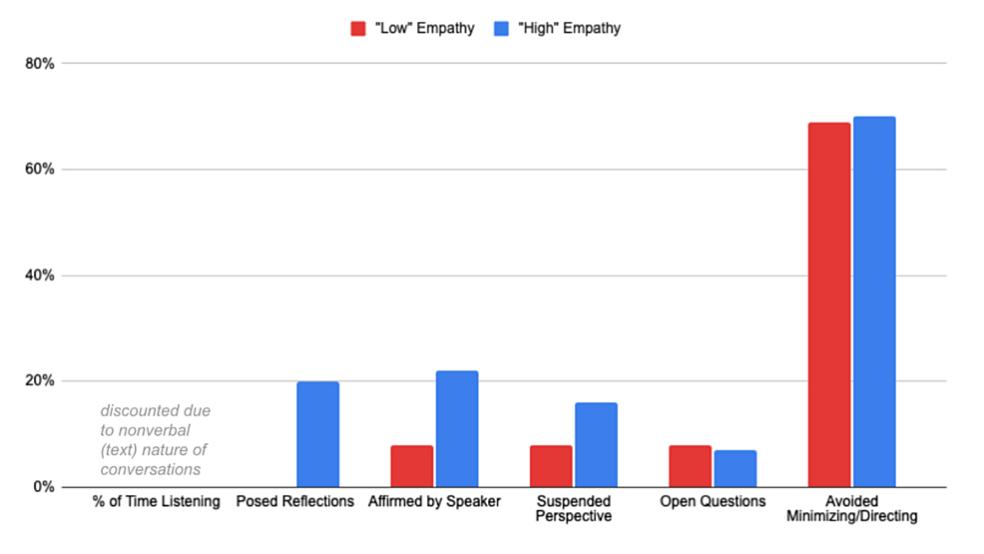
Categories of empathy in “low-” and “high-”ranked conversations within Dataset 1

In addition, the importance of getting affirmations for being on track (+14%) and suspending one's perspective while responding (+8%) also emerged as key factors of high-empathy communicators. By contrast, the use of open questions and avoiding directing or minimizing the conversation remained consistent between high- and low-rated conversations. Because this was the one dataset of text-only evaluations, the category of participants’ “time spent listening” was discounted (see Figure 3).

MINT-member therapists (Dataset 2)

**Figure 4:**
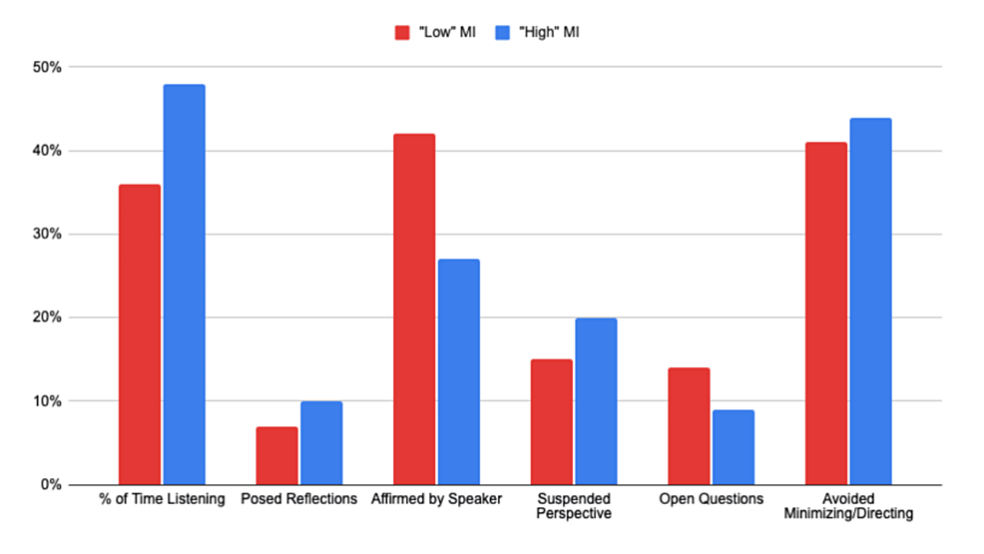
Categories of empathy in “low-” and “high-”ranked conversations within Dataset 2

In conversations evaluated by the 10 MINT-member therapists, the algorithm indicated that time spent listening versus speaking was the most critical factor, showing a 12% increase in listening for conversations rated as "high MI" (see Figure 4). However, other categories presented challenges in distinguishing between high and low MI, with a range of ±5% in high/low MI conversations. Interestingly, getting affirmations from speakers for being on track actually showed a decrease (-15%) in the "high-MI" conversations.

MITI code creators and MI experts (Dataset 3)

The final dataset also presented some distinctive characteristics. Even conversations labeled as "low-quality MI" retained qualities of effective counseling, with consistently high levels of affirmation by speakers (53%) and suspending one's perspective (28-29%). Notably, these categories were approximately ten percentage points higher than Datasets 1 and 2, regardless of high/low empathy labels within those datasets.

**Figure 5:**
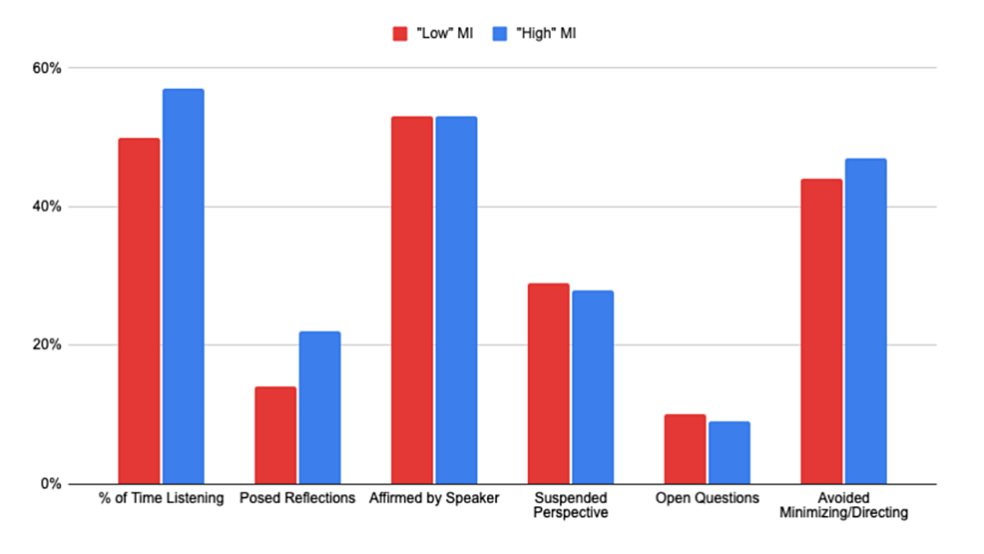
Categories of empathy in “low-” and “high-”ranked conversations within Dataset 3

The Empathy Algorithm's scoring also affirmed two key tenets of MI within Dataset 3: listening and reflecting both showed strong increases (+7% and +8%, respectively) in "high MI"-rated conversations. Avoiding minimizing and directing also increased (3%) in the "high MI” category, albeit only slightly (see Figure 5).

General trends across datasets and comparison to ongoing studies

Across all three datasets, there were smaller changes (±4%) in four of our six categories. While this might suggest that these skills matter less to evaluators of all levels, more recent explorations with our algorithm indicate that different populations may simply emphasize or focus on different specific skills. For example (in a separate ongoing study), when we used the Empathy Algorithm to evaluate conversations of approximately 300 customer service professionals with no counseling backgrounds, skills like open-ended questions were used 20-30% more often than what was demonstrated in these more counselor-focused datasets.

**Figure 6:**
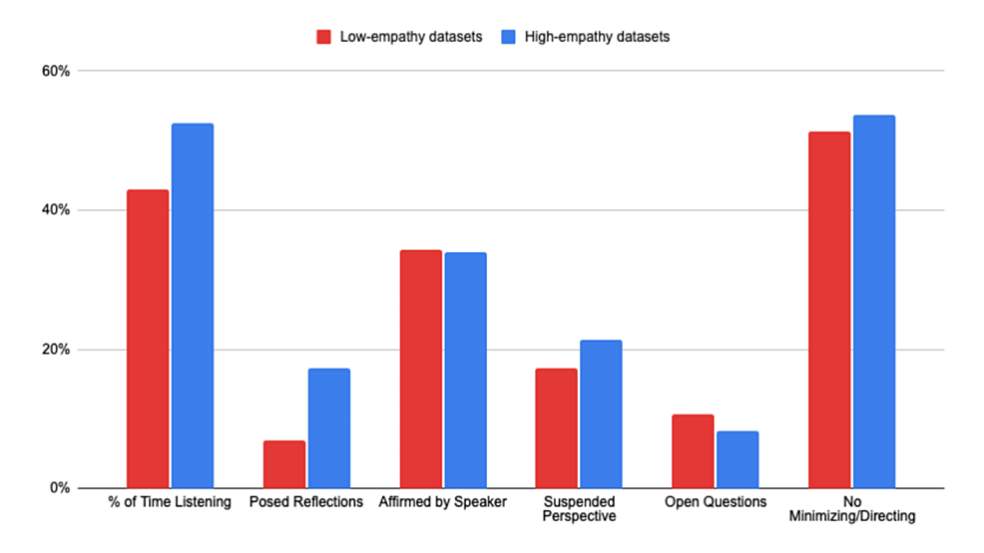
Combined empathy skill categories of “low”- and “high”-ranked conversations from all three datasets

When the deltas for all three datasets were averaged, the two categories from the Empathy Algorithm that consistently indicate “high empathy” - in agreement with all three human evaluator groups - are listening more than speaking and offering reflective statements. These two skills increased between 9% and 10% on average when comparing low- and high-quality empathic conversations. Two other skills, avoiding minimizing or directing the conversation and suspending one’s perspective, also increased on average between 2% and 4%, respectively (see Figure 6).

## Discussion

From multimodal expansion to nuanced classification frameworks, the study not only provides a comprehensive evaluation of the Empathy Algorithm but also outlines the evolving landscape of assessment and training methods in the digital age. This exploration stands as an invitation for researchers and practitioners to contribute to the ongoing discourse, collectively shaping ways to enhance empathic professional communication.

Our exploration into the development and application of the Empathy Algorithm underscores the potential of machine learning in assessing a fundamental yet elusive aspect of human communication - empathy. The convergence of clinical psychology, behavior change expertise, and machine learning algorithms has given rise to tools capable of objectively evaluating empathic communication, offering a valuable resource for people in the caring professions [23,24].

Our algorithm adds to the emerging field of ML-driven conversation-skill assessment and also provides granular insights into what different groups of human evaluators consider to be “good” empathy. Across datasets, our findings illuminate specific features and dimensions of communication that are seen as empathetic, offering valuable guidance for professionals seeking to enhance their empathizing skills.

Our study also reveals the nuanced nature of empathy evaluation, with different datasets exhibiting varied emphases on empathic skills. For non-trained empathetic volunteers, for example, the ability to offer reflections was pivotal, emphasizing the importance of one’s ability to make insightful guesses about or extensions of the speaker's intended meaning. Meanwhile, conversations with professional counselors highlighted the significance of time spent listening, showcasing how different empathizing skills may be used more in different contexts.

Furthermore, even “low-MI” conversations conducted by MI experts (Dataset 3) had consistently high levels of speaker affirmations and suspending of the counselor’s perspective, about 10% more on average than the conversations of Datasets 1 and 2. This might suggest that the MI experts who collected and participated in the conversations of Dataset 3 were more skilled at demonstrating effective empathizing as a whole, even while they were role-playing specific "low-quality MI” tendencies, such as not reflecting a participant’s change talk [17]. This may further affirm how different industries and experts likely focus on different skills in their conversations. Continuing to differentiate and study these skill subdivisions represents an area for further research.

Limitations

In the pursuit of advancing the Empathy Algorithm and understanding its applications, it is essential to acknowledge several inherent limitations that shape the scope and interpretation of our findings. As mentioned prior, because the inclusion/exclusion criteria were not fully detailed in some of the original datasets, it must be noted that certain volunteer-selection and sampling processes might have biased these results. Other limitations to consider include the following:

Modalities and Nuances

Dataset 1 presented a unique challenge as it involved the evaluation of text-only interactions, whereas Datasets 2 and 3 relied on transcripts that also had audio/video components. The absence of non-textual cues, such as tone and body language, in Dataset 1 may limit evaluators’ capacity to capture other potential indicators of empathy, including facial expression, gaze, gestures, and response timing [15]. Recognizing that these nuances can significantly impact empathy labels in conversations, this represents an area for further development. Future studies may benefit from incorporating multimodal data to enhance accuracy and broaden its applicability.

Binary Classification

Empathic responses inherently exist on a nuanced spectrum, on which professionals in different fields may choose different emphases [3]. While the global assignment of “high-” or “low-”quality empathy or MI helped our initial evaluation, it also might not show the nuances of individual responses within a conversation. For example, the original compilers of Dataset 2 assigned the “high-quality” and “low-quality” MI labels based directly on the titles of the video clips they were compiling (e.g., “MI-Good example,” “How NOT to do Motivational Interviewing”) and/or descriptions and comments from the professionals within the video conversations [19]. These videos were all from professional therapists and established institutions for MI, and the codes given by MINT evaluators seem to support each label. Yet, it is important to recognize the inherent limits of simple “high” and “low” classifications. Our intention is to use this initial binary to highlight key trends, in the hopes that others will extend this work with a more nuanced spectrum.

Utterance Pairing Limitation

The Empathy Algorithm, in its current state, evaluates one client/counselor utterance pairing at a time. This design choice poses a limitation in scenarios where the assessment of empathic responses requires consideration of the build-up from several back-and-forth exchanges. Responses dependent on contextual nuances developed over multiple interactions may present challenges for the algorithm in accurately assessing empathy.

Language and Cultural Specificity

The Empathy Algorithm is currently tailored for evaluating conversations in English, limiting its applicability to a specific linguistic and cultural context. Expanding the tool's capabilities to encompass other languages and cultural nuances represents a critical avenue for future development. This expansion will not only enhance the algorithm's versatility but also ensure its relevance in diverse communication scenarios.

These limitations, while intrinsic to the current version of the Empathy Algorithm, serve as valuable insights for refining and evolving the model in subsequent iterations.

Future directions and practical implications

When considering directions for future research, assessing the scalability of algorithm-driven empathy education compared to traditional in-person training and coaching will be key. In addition, we hope future studies expand the use of the Empathy Algorithm beyond the confines of behavioral health, into issues relating to customer retention, workplace engagement, and peer-to-peer conversations. Finally, it is worth considering the impact that scalable, algorithmic feedback mechanisms will have on real-time coaching environments. Virtual- and chat-based skill training, augmented by algorithm-driven feedback, represent promising avenues for further research.

Not only is immediate feedback on the so-called "soft skills" useful for trainees, but it also represents significant time savings on the part of those who traditionally would listen to and grade conversational skill-practice by hand. Using machine-learning tools like the Empathy Algorithm also helps reduce the subjectivity that can be present in even the best of human evaluations. As a practical recommendation, current and future tools like this allow for precise empathic assessments, the objective targeting of improvement areas, and the facilitating of effective communication training at scale.

## Conclusions

The Empathy Algorithm holds promise as a valuable tool for professionals in diverse fields where effective communication is paramount. From counseling and healthcare to customer service and beyond, the ability to objectively assess and enhance empathic skills through machine learning algorithms opens avenues for improving client outcomes, fostering deeper connections, and refining communication practices.

As we navigate the evolving landscape of technology-assisted empathy assessment, our commitment to transparency and knowledge-sharing remains unwavering. We invite fellow researchers and practitioners to explore, replicate, and contribute to the evolution of this work, collectively advancing our understanding of empathy in the digital age.

## References

[1] Wu Z, Balloccu S, Reiter E, Helaoui R, Recupero DR, Riboni D (2023). Are experts needed? On human evaluation of counselling reflection generation. Proceedings of the 61st Annual Meeting of the Association for Computational Linguistics (Volume 1: Long Papers).

[2] Tanana M, Hallgren KA, Imel ZE, Atkins DC, Srikumar V (2016). A comparison of natural language processing methods for automated coding of motivational interviewing. J Subst Abuse Treat.

[3] Elliott R, Bohart AC, Watson JC, Greenberg LS (2011). Empathy. Psychotherapy Relationships That Work: Evidence-Based Responsiveness.

[4] Elliott R, Bohart AC, Watson JC, Greenberg LS (2011). Empathy. Psychotherapy (Chic).

[5] Burns DD, Nolen-Hoeksma S (1992). Therapeutic empathy and recovery from depression in cognitive-behavioral therapy: A structural equation model. J Consult Clin Psychol.

[6] Miller W, Baca L (1983). Two-year follow-up of bibliotherapy and therapist-directed controlled drinking training for problem drinkers. Behavior Therapy.

[7] Moyers TB, Houck J, Rice SL, Longabaugh R, Miller WR (2016). Therapist empathy, combined behavioral intervention, and alcohol outcomes in the COMBINE research project. J Consult Clin Psychol.

[8] Watson JC, McMullen EJ, Rodrigues A, Prosser MC (2020). Examining the role of therapists' empathy and clients' attachment styles on changes in clients' affect regulation and outcome in the treatment of depression. Psychother Res.

[9] Campbell RG, Babrow AS (2004). The role of empathy in responses to persuasive risk communication: overcoming resistance to HIV prevention messages. Health Commun.

[10] Fischer D, Moyers T (2014). Is there an association between empathic speech and change talk in motivational interviewing sessions?. Alcohol Treat Q.

[11] Ellis JD, Grekin ER, Beatty JR (2017). Effects of narrator empathy in a computer delivered brief intervention for alcohol use. Contemp Clin Trials.

[12] Batson C (2009). These things called empathy: eight related but distinct phenomena. The Social Neuroscience of Empathy.

[13] Imel ZE, Pace BT, Soma CS (2019). Design feasibility of an automated, machine-learning based feedback system for motivational interviewing. Psychotherapy (Chic).

[14] Tanana MJ, Soma CS, Srikumar V, Atkins DC, Imel ZE (2019). Development and evaluation of clientbot: patient-like conversational agent to train basic counseling skills. J Med Internet Res.

[15] Xiao B, Huang C, Imel ZE, Atkins DC, Georgiou P, Narayanan SS (2016). A technology prototype system for rating therapist empathy from audio recordings in addiction counseling. PeerJ Comput Sci.

[16] Klonek FE, Quera V, Kauffeld S (2015). Coding interactions in Motivational Interviewing with computer-software: What are the advantages for process researchers?. Computers in Human Behavior.

[17] Miller WR, Rollnick S (2023). Motivational interviewing: helping people change and grow. https://www.guilford.com/books/Motivational-Interviewing/Miller-Rollnick/9781462552795.

[18] Montiel-Vázquez EC, Ramírez Uresti JA, Loyola-González O (2022). An explainable artificial intelligence approach for detecting empathy in textual communication. Appl Sci.

[19] Wu Z, Balloccu S, Kumar V, Helaoui R, Reiter E, Recupero DR, Riboni D (2022). Creation, analysis and evaluation of annomi, a dataset of expert-annotated counselling dialogues. Proceedings of the ICASSP 2022-2022 IEEE International Conference on Acoustics, Speech and Signal Processing (ICASSP), Singapore, 23-27 May.

[20] Moyers TB, Martin T, Manuel JK, Hendrickson SM, Miller WR (2005). Assessing competence in the use of motivational interviewing. J Subst Abuse Treat.

[21] Welivita A, Pu P (2022). Curating a large-scale motivational interviewing dataset using peer support forums. Proceedings of the 29th International Conference on Computational Linguistics. International Committee.

[22] Elliott R, Bohart A, Larson D, Muntigl P, Smoliak O (2023). Empathic reflections by themselves are not effective: meta-analysis and qualitative synthesis. Psychother Res.

[23] Casas J, Spring T, Daher K, Mugellini E, Khaled OA, Cudré-Mauroux P (2021). Enhancing conversational agents with empathic abilities. Proceedings of the 21st ACM International Conference on Intelligent Virtual Agents (IVA).

[24] Sharma A, Lin IW, Miner AS, Atkins Atkins, DC DC, Althoff T (2023). Human-AI collaboration enables more empathic conversations in text-based peer-to-peer mental health support. Nat Mach Intell.

